# Gastrodin improves nerve cell injury and behaviors of depressed mice through Caspase‐3‐mediated apoptosis

**DOI:** 10.1111/cns.14444

**Published:** 2023-08-31

**Authors:** Hongyan Pei, Heping Shen, Jinhao Bi, Zhongmei He, Liping Zhai

**Affiliations:** ^1^ College of Chinese Medicinal Materials Jilin Agricultural University Changchun China; ^2^ Department of Neurology The Second Affiliated Hospital of Jiaxing University Jiaxing China

**Keywords:** behavioral science, Caspase‐3, depression, gastrodin, network pharmacology

## Abstract

**Aim:**

We investigated the effects and target of gastrodin (GAS) for treating depression through network pharmacology combined with experimentation.

**Methods:**

The therapeutic target and signal of GAS for depression were analyzed by network pharmacology. Depression in mice was mimicked with a chronic unpredictable mouse stress (CUMS) model. Through open field, elevated plus maze, forced swimming, and tail suspension tests, the effects of GAS on the CUMS mice behaviors were examined, and the levels of neurotransmitters were detected. The histopathological changes were assayed by H&E and IHC staining, and the protein expressions were detected by Western blotting. Small molecule‐protein docking and molecular dynamics experiments were conducted to simulate the binding mode between GAS and Caspase‐3.

**Results:**

Network pharmacological analysis revealed that Caspase‐3 was the action target of GAS. GAS could improve depression‐like behaviors in CUMS mice, elevate their neurotransmitter levels, ameliorate their nerve cell injury, and inhibit their Caspase‐3 expression. After knocking out Caspase‐3, the effects of GAS were inhibited. Molecular dynamics simulation and small molecule‐protein docking found that GAS bound to Caspase‐3 at SER25, inhibiting the maturation and activation of Caspase‐3.

**Conclusion:**

We find that GAS can act as a Caspase‐3 inhibitor, which improves depression‐like behaviors and nerve cell injury in CUMS mice by inhibiting Caspase‐3‐mediated apoptosis.

## Graphical abstract

Gastrodin can act as a Caspase‐3 inhibitor, which improves depression‐like behaviors and nerve cell injury in CUMS mice by inhibiting Caspase‐3‐mediated apoptosis.

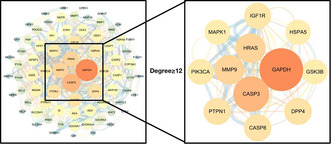

## 1 BACKGROUND

As a mental illness, depression threatens human health seriously.^1^ Drugs currently used for treating depression include tricyclic antidepressants, selective 5‐HT uptake inhibitors, MAOIs, etc.^2^ However, they produce a variety of adverse reactions, making it imperative to find and develop novel drugs.^3^ Rhizoma Gastrodiae, a major herbal medicine used in the clinical treatment of dizziness, vertigo, and other neurological disorders, has diverse pharmacological functions, including sedation, anti‐depression, anti‐anxiety, and anti‐oxidation.^4^, ^5^ To date, there have been many reports concerning anti‐depressive treatment with Rhizoma Gastrodiae, although its exact pharmacological basis and action target remain unclear.^6^, ^7^

It has been found that gastrodin (GAS), one of the important constituents in Rhizoma Gastrodiae, may inhibit depression through neuroprotective effect.^8^ According to extant findings, brain neurons can be damaged by a variety of stressors, including neurotoxins, inflammation, and stress responses.^9^ Neurotoxins refer to toxic substances that damage nerve tissues. GAS has been discovered to inhibit nuclear neurotoxins and elevate the expressions of proteins associated with neural repair and protection.^10^ Animal experiments have demonstrated that the ethanol, aqueous, and ethyl acetate extracts of Rhizoma Gastrodiae, GAS, 4‐hydroxybenzyl alcohol, and vanillin can all improve depression in mice prominently,^11^ which exert antidepressant effects primarily through multiple pathways like monoamine neurotransmitter, antioxidant, anti‐inflammatory, BDNF, and HPA axis regulations.^12^, ^13^ Thus, Rhizoma Gastrodiae contains and involves diverse antidepressant substances and mechanisms, which deserves in‐depth research. Based on prior reports, we used network pharmacology to predict the anti‐depressive target of GAS and made further confirmation.

## 2 MATERIALS AND METHODS

### 2.1 Mouse grouping

Male C57BL/6J mice aged 6–8 weeks were used to conduct animal experiments, which complied with the animal ethics and welfare regulations. Chronic unpredictable mouse stress (CUMS) model was adopted as the mouse depression model. The mice were applied with seven kinds of stimuli. Every day, 1 stimulus was randomly given and identical stimuli were not given for 3 consecutive days, so as to ensure that the mice were unpredictable. After continued stimulation for 4 weeks (28 days), a mouse model of CUMS‐induced depression was established.^14^, ^15^ We divided the wild‐type mice into Control, CUMS, and GAS groups. In the GAS groups, GAS was intragastrically administered into mice at 10 mg/kg (GAS‐L) and 20 mg/kg (GAS‐H) once daily for 28 consecutive days, which was implemented concurrently with the CUMS application. During the mechanism research, Caspase‐3‐knockout mice (Cyagen Biosciences) were used for experiments, which were divided into Control‐KO, CUMS‐KO and GAS+CUMS‐KO groups. In the GAS group, 20 mg/kg GAS was intragastrically administered once daily, while the CUMS operation was the same as above. The intervention was also continued for 28 days.

### 2.2 Elevated plus maze

The mice were placed in the central area of the maze toward the open‐arm direction, and let move freely for 6 min. Their behaviors were recorded with a video tracking system, and their activities within 5 min were analyzed via EthoVision software.

### 2.3 Open field test

The open field consisted of a square area (40 × 40 cm) surrounded by continuous opaque walls. The mice were placed in the same position in the open field center. Their activities were monitored for 10 min and analyzed via EthoVision software. The total movement distance of each mouse in the open field was estimated.

### 2.4 Forced swimming test

A glass bottle 11 × 30 cm in diameter was added with 22–25°C water to 15 cm. On the 1st day, all the mice were forced to swim for 15 min. On the 2nd day, the mice were placed in the same environment for 6 min, and their activity in the subsequent 4 min was monitored. The proportion of immobility time for each mouse was calculated via EthoVision.

### 2.5 Tail suspension test

The end of each mouse tail (2 cm from the tail tip) was taped to the hanging rod (30 cm from the ground), and activity monitoring was carried out for 6 min with a video tracking system. The proportion of immobility time was calculated via EthoVision, where immobility was defined as absence of voluntary or escape‐oriented movement. After completion of the test, the mice were replaced into the cages.

### 2.6 Neurotransmitter detection

Mouse brain tissues were isolated, cleaned twice with PBS, ground to powder with liquid nitrogen, and lysed on ice with 1 mL of NP‐40 buffer (Beyotime Biotechnology) for 30 min. The supernatant protein solution was collected, the levels of 5‐HT, DA, and NE were determined by high‐performance liquid chromatography (HPLC) and calculated by the standard curve method.

### 2.7 H&E

The mouse brain tissues were fixed in 4% formaldehyde for 24 h, dehydrated with gradient alcohol, permeabilized with xylene, soaked in liquid paraffin for 90 min, and then sectioned. After treating with xylene and anhydrous alcohol, the tissue sections were stained using hematoxylin for 8 min, treated with natural water and hydrochloric acid alcohol, and then stained using eosin for 15 min, followed by treatment with gradient alcohol. Finally, the sections were treated with xylene and sealed with neutral gum.

### 2.8 Immunohistochemical (IHC) staining

Tissue sections were treated with reference to H&E procedure, blocked in 5% serum for 30 min, added with TBST‐diluted Bax (Abcam) and incubated at 4°C overnight. After thrice washing with PBS, the sections were visualized with peroxidase substrate kit (Abcam).

### 2.9 Western blotting

Tissue proteins were extracted with reference to ELISA procedure and quantified by BCA assay. After SDS‐PAGE gel electrophoresis, the membranes were transferred at 300 mA, blocked with 5% skim milk powder for 2 h, and then incubated with TBST‐diluted monoclonal primary antibody (Abcam), and further with HRP‐labeled goat anti‐rabbit secondary antibody (Abcam). Following incubation, chemiluminescent immunoassay was performed, and the optical density was analyzed via Image Pro‐Plus 6.0.

### 2.10 Statistical methods

All measurement data were expressed as $\bar{x}$ ± SD, and processed using SPSS 17.0. After homogeneity of variance test, two groups of data were compared by two‐independent samples *t*‐test, while three or more groups of data were analyzed by one‐way ANOVA. Subsequent inter‐group pairwise comparisons were made by the LSD method. All of the above tests were two‐sided, and differences were considered statistically significant when *p* < 0.05.

## 3 RESULTS

### 3.1 Network pharmacological analysis of GAS for depression treatment

**FIGURE 1:**
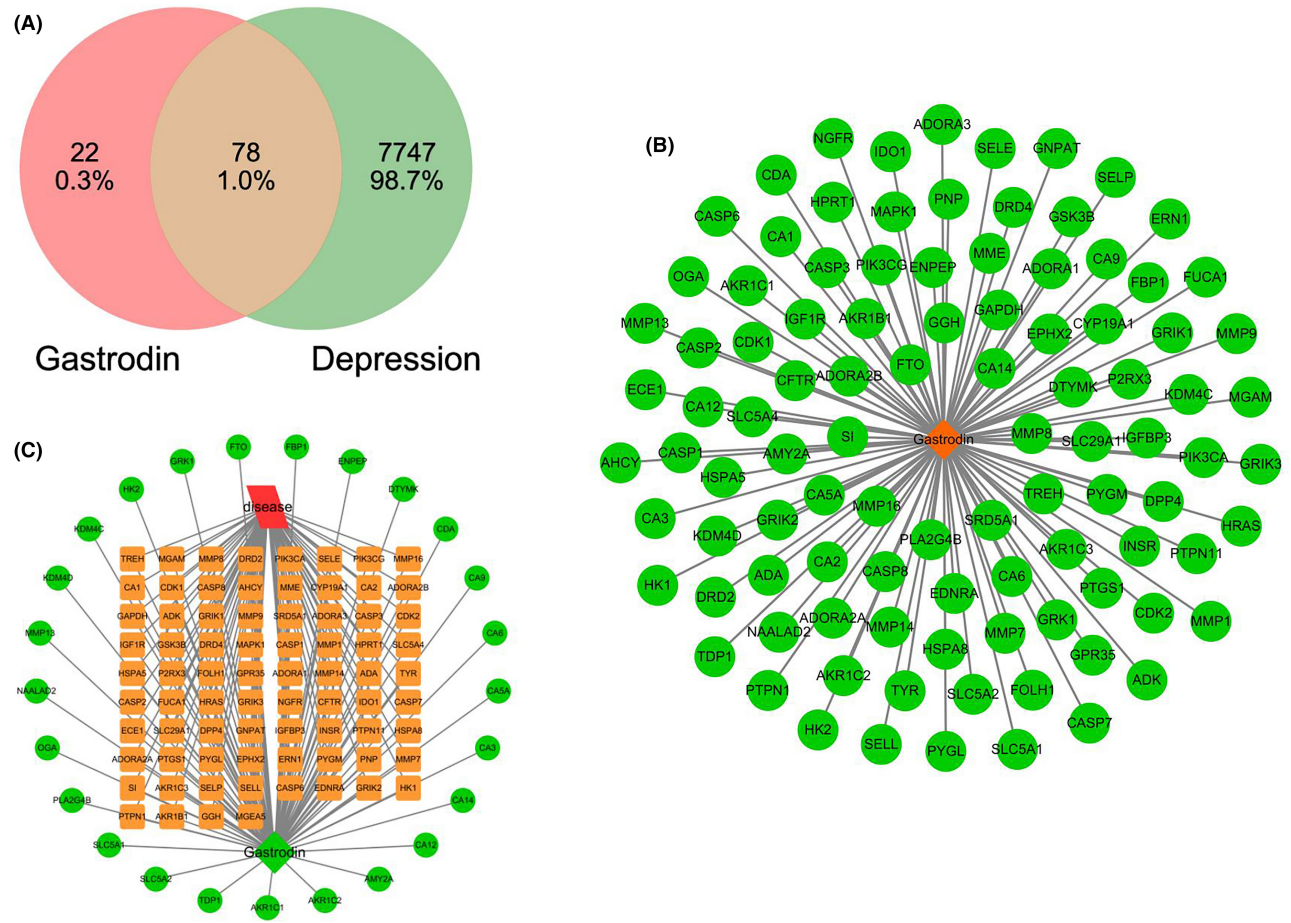
Target analysis of gastrodin (GAS) for treating depression. A: After screening intersections between the two, there were 78 potential targets for GAS. (B, C): Network pharmacological results of GAS targets.

**FIGURE 2:**
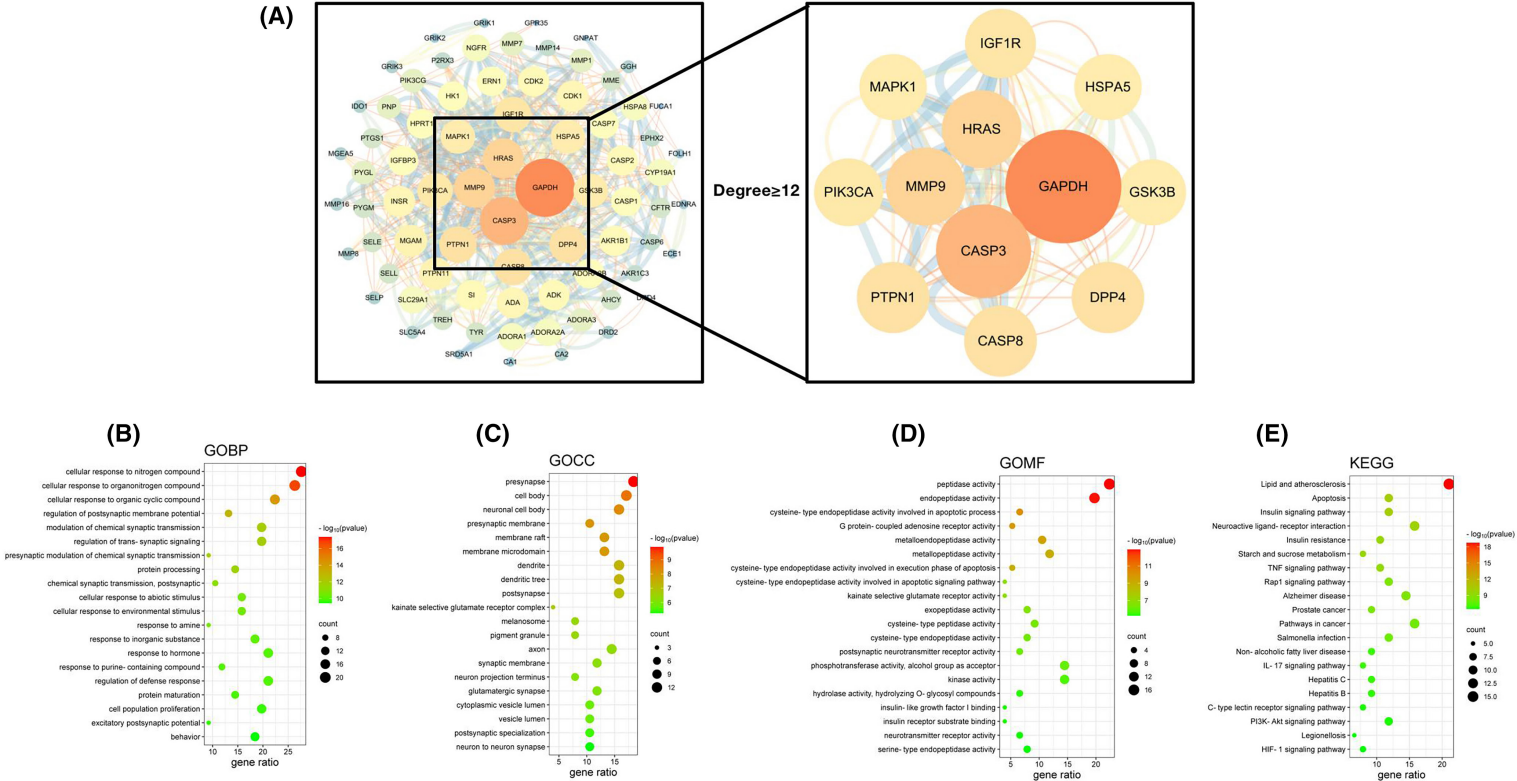
Network pharmacological analysis between gastrodin (GAS) and depression treatment. (A) Scoring results showed that HRAS, MMP9, and Caspase‐3 could serve as the dominant targets of GAS for treating depression. (B–E) Results of GO and KEGG analyses.

We analyzed the targets of GAS and depression treatment, finding that 78 targets could serve as the potential targets of GAS, which were displayed through network pharmacological analysis (Figure 1A–C). Scoring of binding targets revealed that HRAS, MMP9, and Caspase‐3 could be explored as the dominant targets of GAS for depression treatment (Figure 2A). According to the Go and KEGG analyses, GAS was associated with apoptosis and oxidative stress signals (Figure 2B–E).

### 3.2 GAS improved the depressive behaviors and mechanism in CUMS mice

**FIGURE 3:**
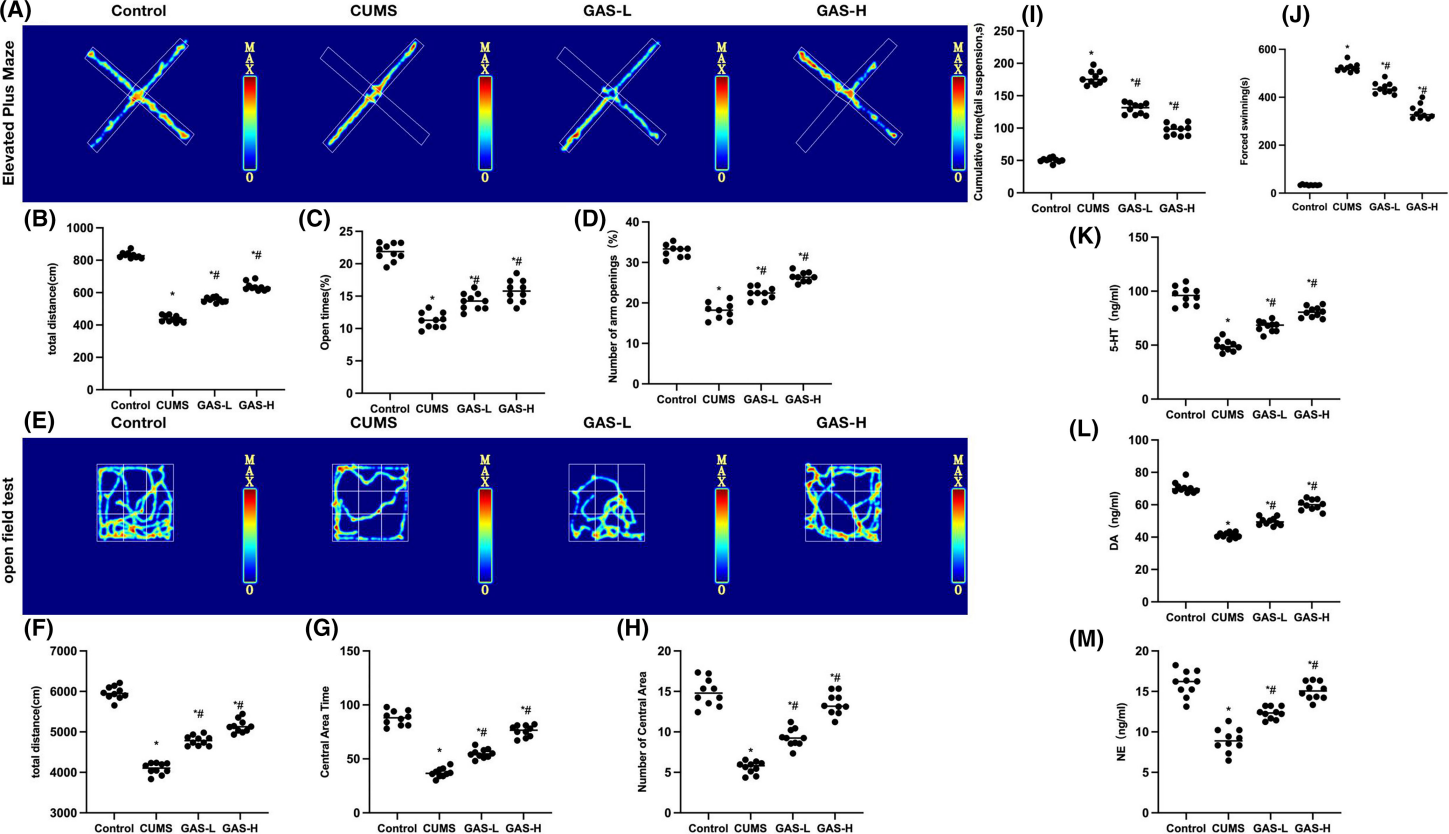
Effects of gastrodin (GAS) on the chronic unpredictable mouse stress (CUMS) mouse behaviors. (A–D): Elevated plus maze (*n* = 10), GAS could dose‐dependently extend the mouse movement distance, and prolong their time and frequency of open arm entries. (E–H) Open field test (*n* = 10), GAS could prolong the total movement distance of mice, as well as their center movement duration and distance. (I) Tail suspension test (*n* = 10), GAS could shorten the mouse suspension time. (J) Forced swimming test (*n* = 10), GAS could shorten the mouse floating time. (K–M) Neurotransmitter detection (*n* = 10), DA, 5‐HT, and NE levels decreased in the CUMS group, while GAS could elevate these neurotransmitter levels. *p < 0.05 versus control; ^#^ *p* < 0.05 versus CUMS.

According to the results, GAS could improve the depressive behaviors of CUMS mice, elevate their tissue levels of neurotransmitters and inhibit their neuronal damage. This mechanism of action worked by inhibiting the Caspase‐3‐mediated apoptosis. Elevated plus maze results showed evident depression‐like behaviors in the CUMS group, shortened total movement distance, as well as time and frequency of open arm entries. GAS could extend the mouse movement distance, and prolong their time and frequency of open arm entries in a dose‐dependent manner (Figure 3A–D). Open field tests revealed significantly shorter movement distance, center movement duration, and distance of mice in the CUMS group than in the Control. GAS could prolong the total movement distance, as well as center movement duration and distance of mice (Figure 3E–H). As indicated by the tail suspension test, the suspension time in the CUMS group was significantly longer than that in Control, while GAS could shorten the suspension time (Figure 3I). In the forced swimming test, the floating time was significantly longer in the CUMS group than in Control, while GAS could shorten the floating time (Figure 3J). Neurotransmitter detection found that the levels of NE, 5‐HT, and DA decreased in the CUMS group, while GAS could dose‐dependently elevate these levels in tissues (Figure 3K–M).

**FIGURE 4:**
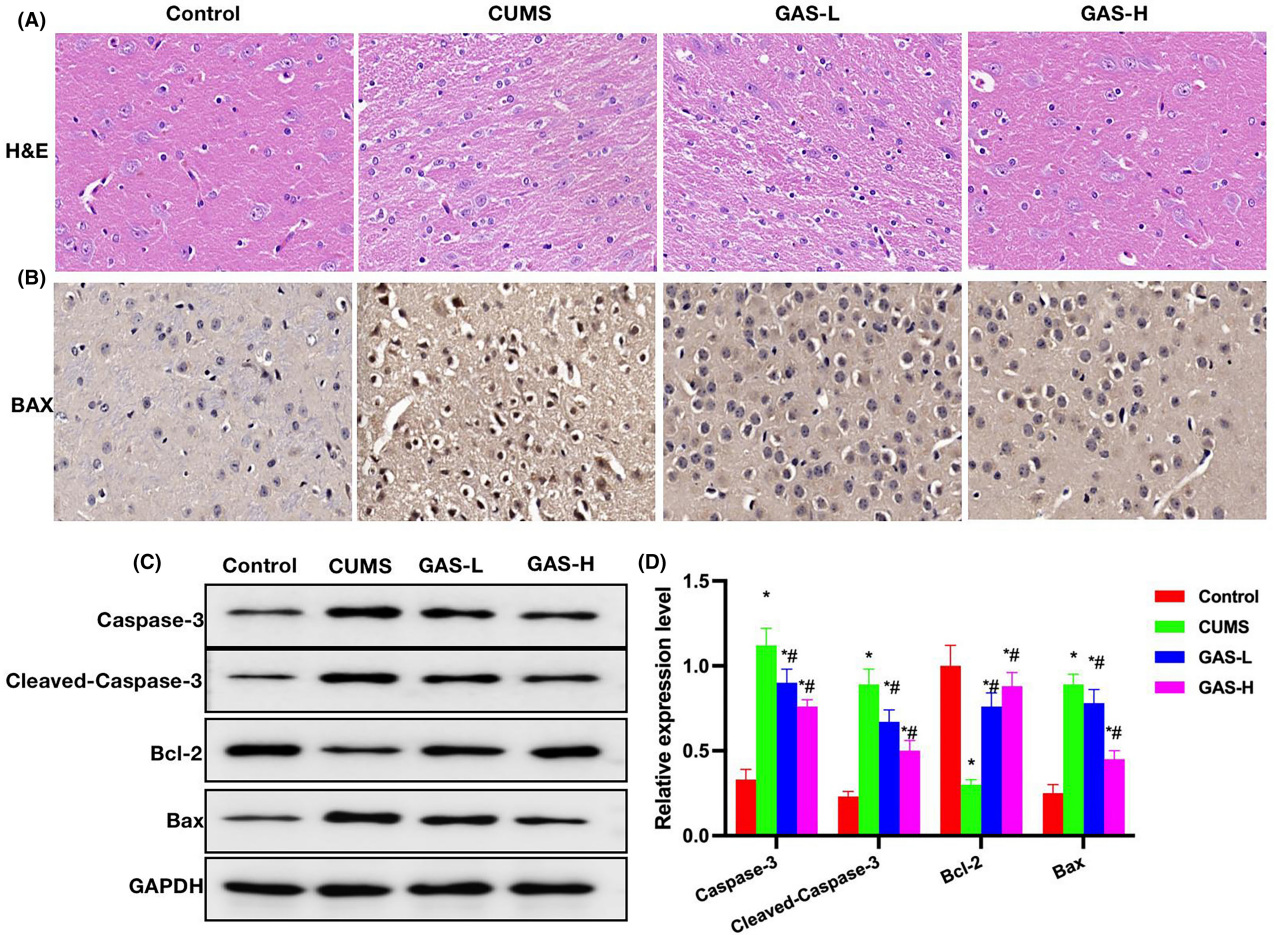
Effects of gastrodin (GAS) on the chronic unpredictable mouse stress (CUMS) mouse histopathology. (A) H&E Nissl staining (*n* = 5), H&E staining revealed nerve cell damage in the CUMS group, while GAS could inhibit the cell damage. (B) IHC staining (*n* = 5), upregulation of Bax protein was noted in the CUMS group, while GAS could lower the Bax expression. (C–D) Relative protein expressions (*n* = 3), upregulation of Caspase‐3, Cleaved‐Caspase‐3, and Bax were noted in the CUMS group, as well as downregulation of Bcl‐2. GAS could lower the Caspase‐3, Cleaved‐Caspase‐3 and Bax levels, and elevate the Bcl‐2 level. *p < 0.05 versus control; ^#^ *p* < 0.05 versus CUMS.

H&E staining revealed occurrence of minor lesions and nerve cell damage in the CUMS group, while GAS could inhibit the cell damage (Figure 4A). As demonstrated by IHC staining results, the level of Bax protein was upregulated in the CUMS group, while GAS could lower the Bax expression (Figure 4B). Relative protein expression assays found that Caspase‐3, Cleaved‐Caspase‐3, and Bax were upregulated in the CUMS group, while Bcl‐2 was downregulated. GAS could lower the levels of Caspase‐3, Cleaved‐Caspase‐3, and Bax, and elevate the level of Bcl‐2 (Figures 4C,D).

### 3.3 Caspase‐3 knockout could inhibit the effects of GAS

**FIGURE 5:**
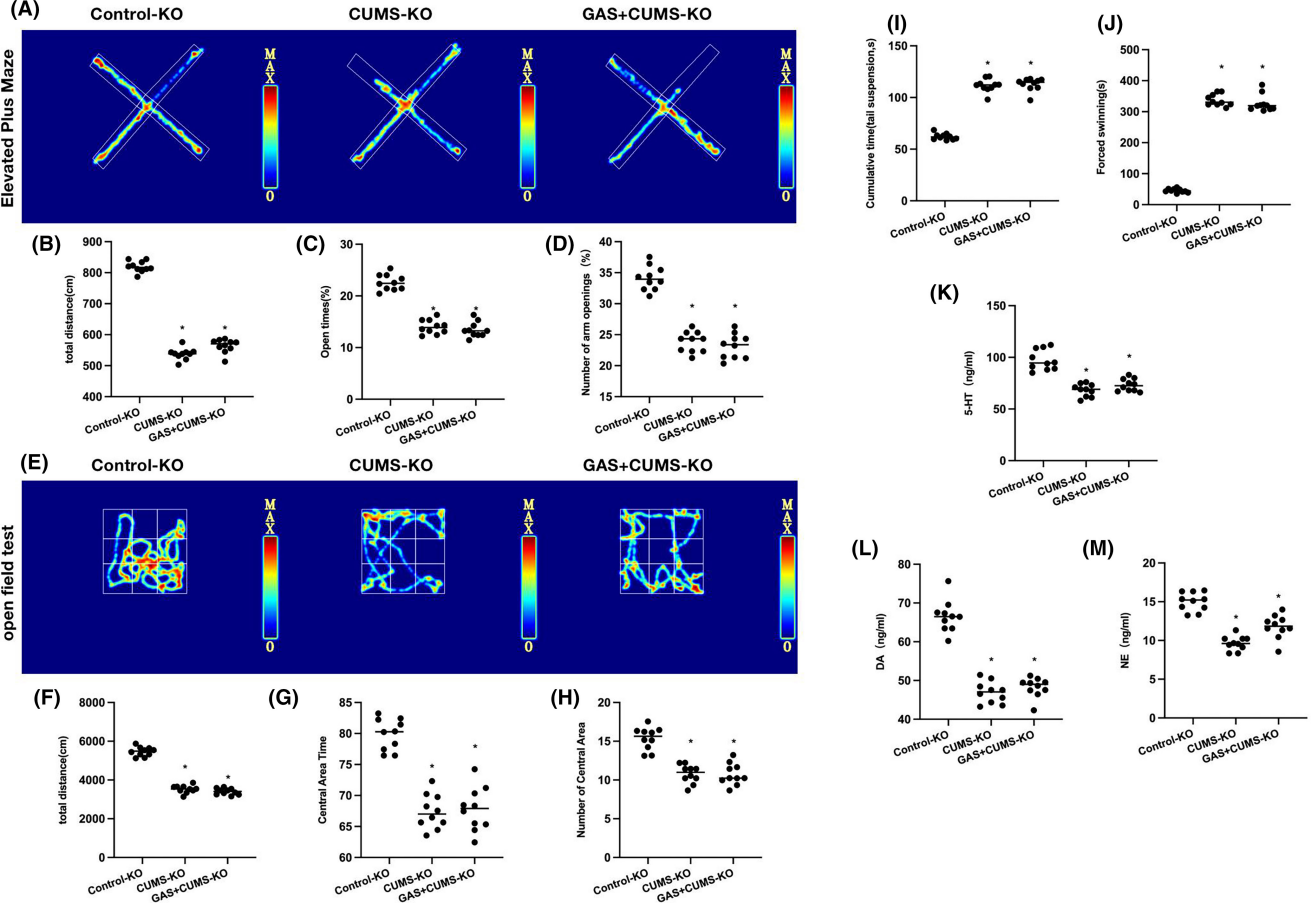
Caspase‐3 knockout could inhibit the effects of gastrodin (GAS). (A–D) Elevated plus maze (*n* = 10), differences in the total movement distance, time, and frequency of open arm entries were insignificant between CUMS‐KO and GAS+CUMS‐KO groups. (E–H) Open field test (*n* = 10), insignificant differences in total movement distance, center movement duration or distance were noted between the CUMS‐KO and GAS+CUMS‐KO groups. (I) Tail suspension test (*n* = 10), suspension time did not differ between the CUMS‐KO and GAS+CUMS‐KO groups. (J) Forced swimming test (*n* = 10), floating time of CUMS‐KO group did not differ from that of GAS+CUMS‐KO group. (K–M) Neurotransmitter detection (*n* = 10), the levels of NE, 5‐HT, and DA did not differ between the CUMS‐KO and GAS+CUMS‐KO groups. *p < 0.05 versus. Control‐KO; ^#^ *p* < 0.05 versus CUMS‐KO.

We found that Caspase‐3 knockout could ameliorate the CUMS‐induced depressive symptoms in mice. However, GAS intervention could not further alleviate the depressive symptoms and produced an insignificant effect on neurotransmitters. According to the elevated plus maze results, the differences in the total movement distance, time, and frequency of open arm entries were insignificant between CUMS‐KO and GAS+CUMS‐KO groups (Figure 5A–D). Open field test found no significant differences in total movement distance, center movement duration, or distance between the CUMS‐KO and GAS+CUMS‐KO groups (Figure 5E–H). Tail suspension test results showed that the suspension time did not differ between the CUMS‐KO and GAS+CUMS‐KO groups (Figure 5I). In the forced swimming test, the floating time of the CUMS‐KO group did not differ from that of the GAS+CUMS‐KO group (Figure 5J). As revealed by neurotransmitter detection, the NE, 5‐HT, and DA levels did not differ between the CUMS‐KO and GAS+CUMS‐KO groups (Figure 5K–M).

**FIGURE 6:**
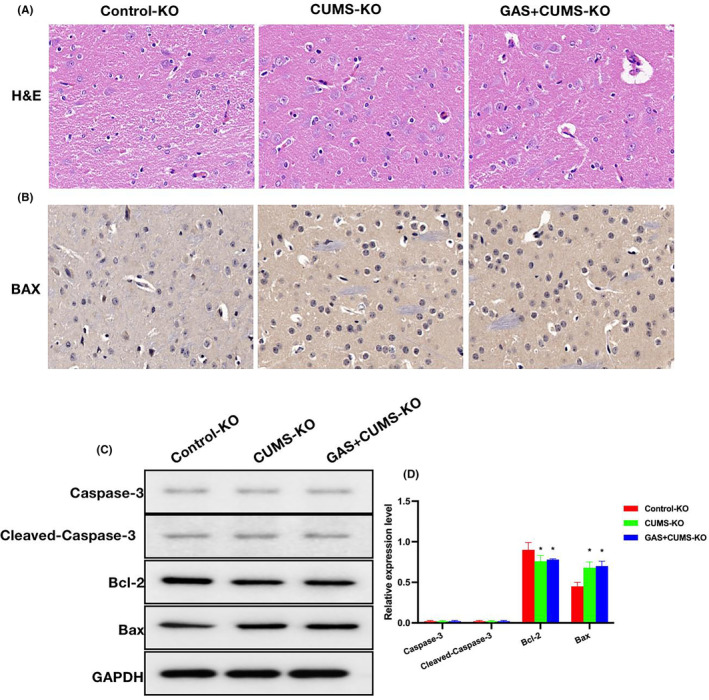
Effects of gastrodin (GAS) on the chronic unpredictable mouse stress (CUMS) mouse histopathology after Caspase‐3 knockout. (A) H&E staining (*n* = 5), difference in nerve cell damage was insignificant between CUMS‐KO and GAS+CUMS‐KO groups. (B) IHC staining (*n* = 5), difference in Bax level was insignificant between CUMS‐KO and GAS+CUMS‐KO groups. (C, D) Relative protein expressions (*n* = 3), Caspase‐3 and Cleaved Caspase‐3 were almost not expressed, while the inter‐group differences in Bax and Bcl‐2 expressions were insignificant. *p < 0.05 versus Control‐KO; ^#^ *p* < 0.05 versus CUMS‐KO.

H&E staining found insignificant difference in nerve cell damage between CUMS‐KO and GAS+CUMS‐KO groups (Figure 6A). IHC staining revealed that the expression level of Bax did not differ significantly between the CUMS‐KO and GAS+CUMS‐KO groups (Figure 6B). During the protein assays, almost no expression of Caspase‐3 or Cleaved Caspase‐3 was noted, while the expressions of Bax and Bcl‐2 differed insignificantly between the two groups (Figure 6C,D).

### 3.4 Dynamics analysis between GAS and Caspase‐3

**FIGURE 7:**
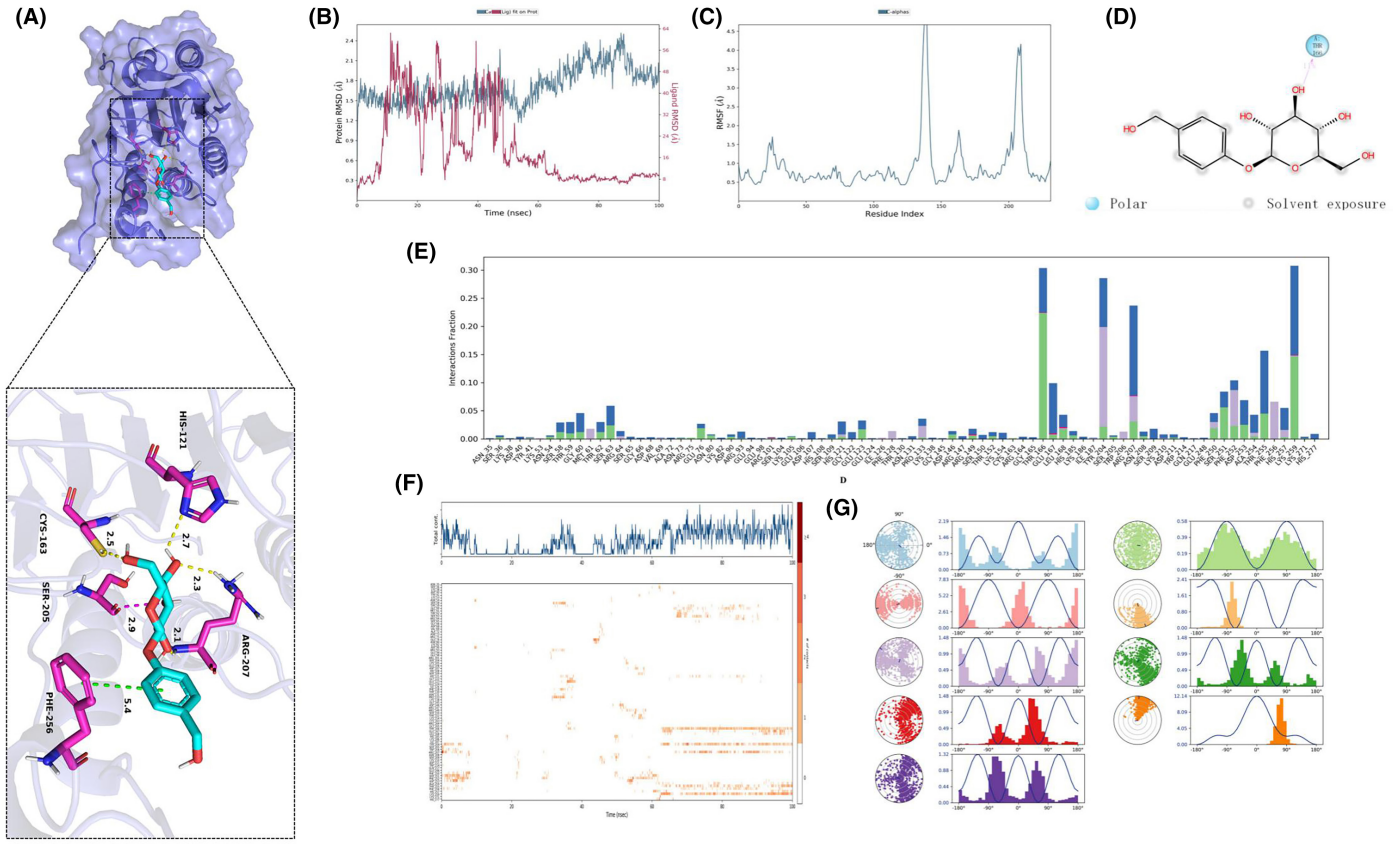
Binding analyses between gastrodin and Caspase‐3. (A) Small molecule‐protein docking; (B–G) Molecular dynamics analysis.

Through small molecule‐protein docking, we found that GAS bound to the Caspase‐3 ARG‐207 site, inhibiting the Pro‐Caspase‐3 cleavage and the Cleaved‐Caspase‐3 formation (Figure 7A). Dynamics analysis of GAS binding to Caspase‐3 (Figure 7B–G).

## 4 DISCUSSION

The basic clinical manifestation of depression, which is a kind of affective mental disorder, is the syndrome of persistent low mood, abnormal thinking, and behavior, and patients with depression are prone to low self‐esteem, black mood, pessimism, and even suicidal tendencies and behaviors.^16^, ^17^ Depression is associated with factors like unemployment, poverty, learning, emotion, and frustration. With the development of society and the accelerating pace of life, its incidence has been rising year by year, becoming a common and frequently occurring disease.^18^ The pathogenesis of depression is complex, which may be related to low psychological and mental activities resulting from the decrease of brain neurotransmitters,^19^ as well as to the elevation of inflammatory cytokines caused by hormonal abnormalities. The injury of nerve cells is an important pathological mechanism inducing depression,^20^, ^21^ and the apoptosis signaling mediated by Caspase‐3 and Caspase‐9 is one of the major mechanisms of nerve cell death.^22^ Expressions of multiple inflammatory factors can promote the activation of Caspase‐3/9 signaling to induce nerve cell damage.^23^ GAS has been proven to possess potent antioxidant and anti‐inflammatory effects, which has also been reported to have the depression‐ameliorating function.^24^ Research has found that GAS can improve depression‐like behaviors in rats by upregulating the brain‐derived neurotrophic factor (BDNF) expression in their hippocampal astrocytes.^25^ Nevertheless, its exact action target has rarely been reported so far.

Through network pharmacological analysis, we found 7747 targets in depression and, after screening the intersection targets with GAS, 78 potential targets were obtained. Further scoring revealed that GAS was closely associated with targets like MAPK1, IGFR, HSP, and Caspase‐3. Caspase‐3 is the primary target signal mediating the damage of nerve cells. We exploited CUMS to establish a mouse model of depression. According to the results, GAS could improve the symptoms of CUMS mice, and enhance their motor and cognitive abilities. Meanwhile, GAS could elevate the levels of neurotransmitters, which are a kind of transmitter secreted by nerve cells for behavioral regulation. We believed that GAS elevated the neurotransmitter levels after protecting nerve cells. Further analysis revealed that GAS could inhibit the activation of Caspase‐3, lower the expression of Cleaved‐Caspase‐3, promote the expression of Bcl‐2, and decrease the level of Bax, primarily inhibiting the apoptosis signal activation. After knocking out Caspase‐3 in CUMS mice, their depressive symptoms were alleviated. However, GAS could not further improve depression, and produced insignificant effects on the neurotransmitter levels, further suggesting that Caspase‐3 was the only target of GAS. Finally, through small molecule‐protein docking and molecular dynamics simulation, we found that ARG207 was the GAS binding site. After binding to this site, GAS could inhibit the Caspase‐3 cleavage.

## 5 CONCLUSION

We find that GAS is a Caspase‐3 inhibitor, which inhibits the cleavage and maturation of Caspase‐3 after binding to ARG207 site, thereby suppressing the nerve cell apoptosis in depression. GAS treats depression by inhibiting the activity of Caspase‐3 to improve the behaviors in CUMS mice. This is one mechanism whereby GAS treats depression.

## AUTHOR CONTRIBUTIONS

Hongyan Pei are mainly responsible for the operation of the experiment, the acquisition of relevant data; Heping Shen and Jinhao Bi are mainly responsible for literature review, project coordination; Liping Zhai and Zhongmei He are mainly responsible for project development, financial support.

## FUNDING INFORMATION

The Science and Technology planning project of Jiaxing (2023AZ11001).

## CONFLICT OF INTEREST STATEMENT

The authors declare no conflicts of interest.

## Data Availability

The data that support the findings of this study are available from the corresponding author upon reasonable request.
